# The Relationship between Cytokine Profile and Hypertension among the Mercury-Exposed Residents of Temirtau Region in Central Kazakhstan

**DOI:** 10.18502/ijph.v49i8.3894

**Published:** 2020-08

**Authors:** Lyazzat SHINETOVA, Almira AKPAROVA, Saulemai BEKEYEVA

**Affiliations:** Department of General Biology and Genomics, L.N. Gumilyov Eurasian National University, Astana, 010008, Kazakhstan

**Keywords:** Mercury, Cytokines, Hypertension, Kazakhstan

## Abstract

**Background:**

Mercury is a common environmental contaminant and it is also harmful to human health. Among reported toxicities, its harmful effect on hypertension is poorly documented. In Kazakhstan, Temirtau city has been reported to have a high level of mercury contamination from an acetaldehyde production factory. Therefore, we aimed to investigate the association between serum profile of cytokines and the development of hypertension among the exposed citizens.

**Methods:**

We selected 81 individuals for study, out of them, 41 exposed ones suffered hypertension and 40 – unexposed healthy controls in villages Chkalovo, Samarkand, Gagarinskoye, Tegiszhol, Rostovka in 2016. Mercury content in urine was studied by inversion voltammetry. Cytokine levels of IL-2, IL-6, IL-10 and TNF-α were determined by ELISA.

**Results:**

Mercury-exposed citizens, especially those with hypertension, had significantly higher concentrations of inflammatory cytokines TNF-α, IL-2, IL-6 and anti-inflammatory cytokine IL-10 as compared to the unexposed population. The dependence of the mercury level in urine on IL-2 content was also detected. Therefore, chronic low doses of exposure to mercury were associated with an increase in serum levels of immune markers and with the increased risk of hypertension.

**Conclusion:**

The presence of mercury in the body probably affected the expression of interleukin-2, one of the main cytokines that coordinate immune response.

## Introduction

Mercury is an environmental pollutant which can exist in three basic forms: elemental, inorganic and organic (1). Unfortunately, mercury is poisonous and toxic, depending on the exposure form and the route of exposure (2). In addition, exposure to mercury can cause central nervous system toxicity, perturbation of the immune system and changes in gene and enzyme expressions (3–6).

Elemental mercury, also known as metallic mercury, in vapor form is readily absorbed through the lungs and can cause toxicity (3, 2). It can pass through the cell membranes, as well as blood-brain and placental barriers due to its diffusion capacity. Inorganic mercury, a divalent compound, is the toxic species that readily deposits in human tissues after converting from other forms. Organic mercury, represented by methylmercury and ethylmercury, enters the human body via consumption of fish products and marine mammals, and the use of mercury-containing vaccines (3, 5).

Mercury pollution of the Nura River in Central Kazakhstan arose as a result of the discharge of wastewater from acetaldehyde production. The occurrence of mercury in the river channel is mainly due to the fly ash from the electrical power stations. The maximum concentration of mercury was found in Temirtau city located 15 km below the pollution source. The largest contamination site, about 75 km long, was located downstream and was estimated to contain about 9.4 tons of mercury (7, 8).

In 2005, a survey was carried out among the population who lived around the mercury exposure territory of Temirtau city. Fishermen of over 45 yr old had the highest level of mercury in their hair. A significant association between hair concentration of mercury and the frequency of consumption of river fish was reported (9).

Because of the pollution and health concerns, the mercury-contaminated areas along the Nura River were cleaned up with the funding from the World Bank and the Republic of Kazakhstan in between 2007 and 2011. A subsequent evaluation after the clean-up showed that mercury levels in the soil and the sediments of the Nura River had reached safe limits (10).

In 2013 and 2014, a systematic study on heavy metals in the Nura River and its environs was conducted. The levels of mercury, methylmercury, polychlorinated biphenyls and organochlorine pesticides in the sediments and the soil, as well as biotic samples (fish, eggs) were evaluated (11). The study revealed that the excessive concentrations of mercury in sediments and soils could pose health risks for populations who consumed contaminated crops and fish from the area. In addition, high concentrations of polychlorinated biphenyls were identified in the samples of sediments and eggs taken from several settlements.

The long-term exposure to mercury can cause a variety of toxic effects, such as damage to the central nervous, renal, immune, cardiovascular and reproductive systems (2, 3, 12, 13). The dysfunctionality of the cardiovascular system includes ischemic heart disease, hypertension, myocardial infarction and cardiac arrhythmia.

High levels of mercury in hair were associated with atherosclerosis, hypertension and other cardiovascular diseases (6, 14). In addition, the exposure doses of methylmercury for cardiovascular diseases were lower than those for neurotoxicity (5, 14). The question is still open on what the safe exposure doses of mercury are for the general population (6).

Cardiovascular and hypertension effect from mercury exposure can be caused by the activation of oxidative stress and inflammation, the reduction of oxidative defense, thrombosis, the dysfunction of vascular smooth muscles, dyslipidemia, endothelial, mitochondrial and immune dysfunction (5, 13–16). “Specifically, mercury exposure was associated with increased blood pressure and pulse pressure in adult Inuit after considering the effect of nutrients for fish (n-3 fatty acids and selenium) and other factors “(15).

Therefore, one general assumption is that mercury toxicity involves oxidative stress and the activation of the immune system through the mobilization of cytokines (17). However, no studies on immunological effects of mercury exposure had been conducted in Temirtau city to validate the general assumption.

From our recent survey of the population in Temirtau city, we found that many citizens with mercury exposure complained about frequent headaches, fast fatigue, dizziness associated with hypertension. Therefore, our aim for the current study was to determine the impact of mercury exposure on the levels of serum cytokines in patients with hypertension.

## Materials and Methods

### Background and the study population

**Fig. 1:**
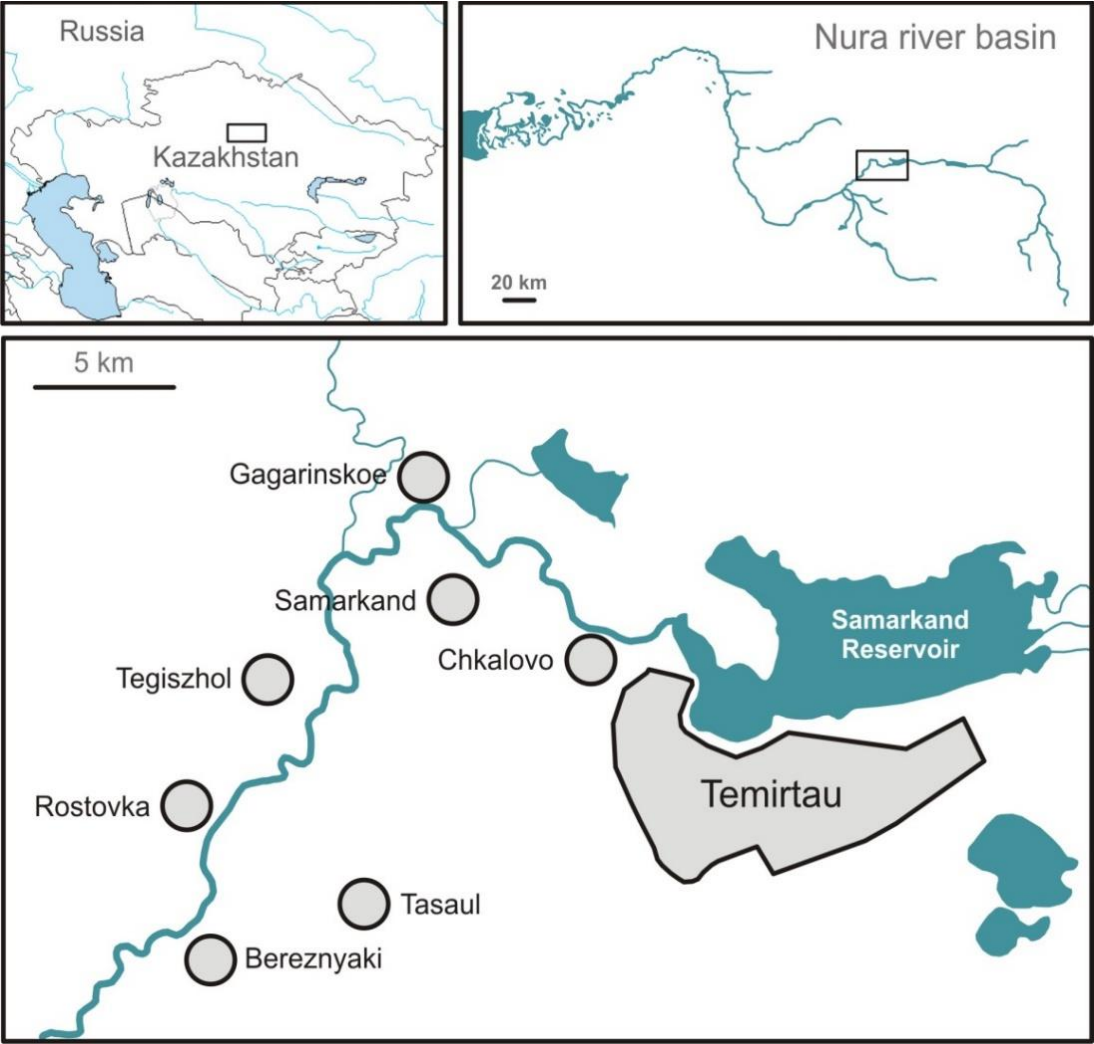
Map of study sites within Kazakhstan

Temirtau city is located in the northern part of Central Kazakhstan. From 1965 up to 1997, the “Karbid” chemical factory was the main source for mercury contamination of the Nura River. Therefore, the sampling of blood and urine was carried out in randomly selected residents who lived within a radius of 70 km from Temirtau city and along the river in the villages of Chkalovo, Samarkand, Gagarinskoe, Tegiszhol, Rostovka for at least 10 years (Fig. 1).

The controls were randomly selected from near-by mercury-free cities and were matched with the exposed residents according to their gender, age and socioeconomic status. The exclusion criterion for the controls was any evidence of hypertension. Both groups were interviewed for personal history and demographic information. In addition, hospital data on hypertension and other cardiovascular diseases were collected.

The samples of blood and urine were taken only from those participants who signed the informed consent forms to contribute to the study. The samples of water from the Nura River, blood and urine were collected from the participants on the same day.

This study was conducted in compliance with ethical principles for medical research set forth in the Declaration of Helsinki (2013), and approved by the Ethics Committee of the Medical University of Astana on Apr 14, 2014.

### Assessment of mercury contamination in water and urine

The water intake was conducted in 20 different locations of this area. The water was collected into plastic bottles (V=500 ml) with twisting lids. Prior to analysis, samples were stored in cold and dark conditions.

The mercury content of the water samples was analyzed in the monitoring laboratory of “Bio-medpreparat” scientific and analytical center (Stepnogorsk) by the cold-vapor atomic absorption method using FIAS-100 (Flow Injection for Atomic Spectroscopy System, Perkin Elmer) and AAnalyst 300 (Atomic Absorption Spectrometer, Perkin Elmer).

Water samples were prepared with pH adjustment, addition of H_2_SO_4_, HNO_3_, potassium permanganate solution and potassium persulfate. Analysis and reduction solutions were injected to the test tube with a barometer lowered to the bottom and an upper discharge tube, and air was introduced by the gas bubbler. The recovered mercury entrained by the current of air passed through the drop catcher and desiccant, being freed from possible sources of interference, and entered to the absorption cuvette. Then, light absorption was measured at 253.7 nm.

The mercury content was determined in urine by the method of inversion voltammetry. About 20–50 ml of urine samples were collected in a sterile plastic collection cups. The samples were immediately delivered to the laboratory to measure the mercury content.

About 1.0–2.0 ml of urine samples were poured to 20–25 ml quartz cup, then nitric acid was added and the samples were incinerated in an electric furnace under the controlled temperature conditions with the addition of nitric acid and hydrogen peroxide prior to ashing. The resulting ash was dissolved in hydrochloric acid. The mercury content was determined by using inversion voltammetry.

### Measurement of serum cytokines

The blood samples were collected in vacutainers with heparin (BD Vacutainer) and kept at room temperature for an hour. Then the serum was collected into separate tubes and stored at −40 °C until ELISA was performed. Serum tumor necrosis factor-α (TNFα), interleukins 2, 6 and 10 (IL-2, IL-6 and IL10) levels were measured in a subgroup, isolated from the general study population. Overall, 68 people (36 mercury-exposed people and 32 unexposed people as control) were randomly selected to measure the level of cytokines. These participants were compared by their age, sex and the status of tobacco smoking. The serum levels of TNF-α, IL-2, IL-6 and IL-10 were determined by an enzyme immunoassay, using reagent kits of Vector-Best company, Novosibirsk, Russia. The results were evaluated on an EIA-Reader spectrophotometer (Micro-planshet Reader (BiolabSystem).

### Statistical analysis

The Mann-Whitney test was used to determine the differences in the parameters, including mercury content in urine, TNF-α, IL-2, IL-6 and IL10 levels in the serum between the exposed and unexposed groups. The data were defined as mean ± SD. Statistical processing of data was carried out using Microsoft Excel and Graph Pad Prism 6. *P*≤0.0125 after Bonferroni correction was considered statistically significant.

## Results

### Characteristics of the population

A summary of the characteristics of the exposed and unexposed populations is shown in Table 1. The average age of the mercury-exposed participants was 54±13.5 yr, while the average age of the unexposed was 53±13.2. No significant differences in the distribution of age, sex, tobacco smoking and socioeconomic status between the groups were found. The exposed group predominantly consisted of retirees (44%), school teachers (12%), former miners (7%), drivers (7%) and house workers (30%). Among all exposed residents, 41 patients with hypertension were selected.

**Table 1:** Characteristics of the study populations

***Variables***	***Mercury-exposed population N=41***	***Control group N=43***	***P-value***
Age, year, Mean ± SD	54±13.5	53±13.2	>0.05
Women, n (%)	18 (43.9)	19 (44.2)	>0.05
Men, n (%)	23 (56.1)	24 (55.8)	>0.05
Body mass index (BMI**)**, n (%)			
<20 kg/m^2^	1 (2.4)	2 (4.7)	
20 – 25 kg/m^2^	3 (7.3)	9 (20.9)	
25 – 30 kg/m^2^	17 (41.5)	23 (53.5)	
> 30 kg/m^2^	20 (48.8)	9 (20.9)	
Smoking, n (%)			
Non-smokers	34 (82.9)	32 (74.2)	>0.05
Smokers	7 (17.1)	11 (25.6)	
Stage 1 hypertension, n (%)	18 (43.9)	-	
Stage 2 hypertension, n (%)	6 (14.6)	-	
Stage 1 hypertension (Hypertensive emergency), n (%)	17 (41.5)	-	
Mercury content in water (Mean ± SD) (μg Hg/L)	4,5±9,8	0,03±0,08	<0.05
Mercury content in urine (Mean ± SD) (μg Hg/L)	2.53 ± 0,9	0.17 ± 0.05	<0.05

### Mercury exposure characteristics

A summary of the mercury concentrations in water and urine is shown in Table 1. The mercury content in the Nura River exceeded the limit of 0.05 μg/l recommended by WHO. In the unexposed control group, the mercury content in water was always less than 0.05 μg/l. Mercury concentrations in the exposed residents were significantly higher as compared to the unexposed residents (*P* <0.05).

The exposed group was subsequently divided into two subgroups, depending on the concentration of mercury in the urine: the first group included the participants with mercury levels below 3.0 μ Hg / L, which represents the 95th percentile for adults (Dye BA). The second group included the participants with the mercury content above the threshold level.

### The content of cytokines in serum

**Fig. 2:**
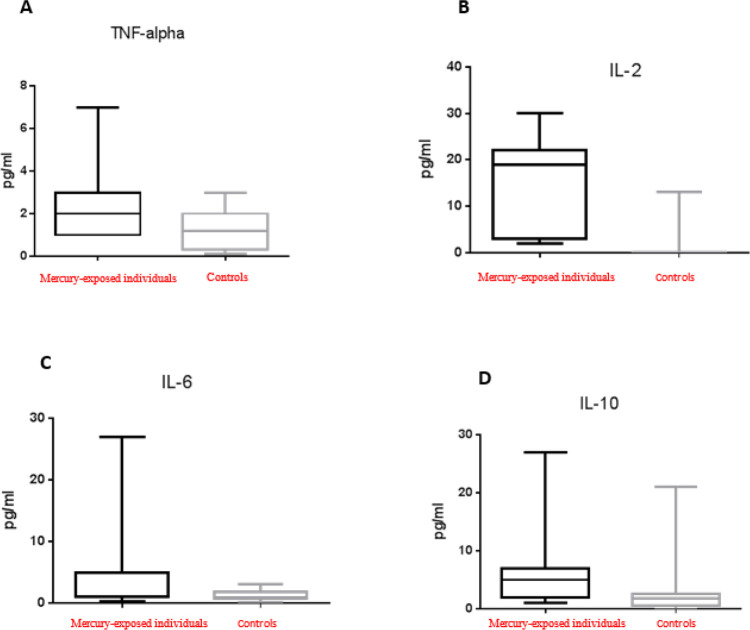
Cytokine levels in the blood serum of mercury-exposed patients with hypertension and control group. Compared to control group patients with hypertension showed high levels of TNF-α (*P*=0.0007) (A), IL-2 (*P*<0.0001) (B), IL-10 (*P*=0.0003) (D) cytokines and a tendency for higher IL-6 (*P*=0.0233)^*^ (C) ^*^P-value 0.05–0.0125 was considered as a tendency after Bonferroni correction

The levels of TNF-α, IL-2, IL-6 and IL-10 in serum were determined in mercury-exposed participants, suffering from hypertension. TNF-α, IL- 2, IL-6 and IL-10 were significantly elevated in the exposed group, compared to the control group (Fig. 2).

In addition, there was a significant change in the IL-2 content, depending on the level of mercury in the urine (*P*<0.0001) (Table 2).

**Table 2:** Serum cytokine measurements according to mercury levels in urine

***Group***	***N***	***Mercury levels in Urine (μg/L): Median (Range)***	***Sex***	***TNF-α***	***IL-2***	***IL-6***	***IL-10***
High Hg	13	3.98 (3.1–5.2)	M=7 F=6	2.33 (1–3)	31.5^****^ (2–102)	0.98 (0.8–1)	4.47 (1–7)
Low Hg	28	1.69 (0.5–2.8)	M=16 F=12	2.0 (1–4)	15.02^****^ (2–27)	1.2 (0.2–5.6)	4.0 (1–10)

All cytokine levels presented in pg/ml, with the median (interquartile range) represented

**** *P*<0.0001

## Discussion

Chronic mercury intoxication causes the neurotoxic effect but alterations in the immune and cardiovascular systems are not well characterized. We studied the markers of immune responses in the mercury-exposed residents with hypertension, living in the north of Central Kazakhstan. The increase of inflammatory cytokines (TNF-α, IL-2, IL-6 and IL-10) in the serum indicates an increase of systemic inflammation and a risk of hypertension for the mercury-exposed population.

Previous studies have shown a strong association between proinflammatory cytokines level and blood pressure in experimental animals and humans. An increase of the IL-6 and TNF-α levels in the blood plasma was observed among the participants with hypertension in comparison with normotensive participants (18–20). In addition, there are data on the interaction of proinflammatory cytokines IL-6 and TNF-a with the regulatory systems of arterial pressure, such as renin-angiotensin and sympathetic nervous systems. Furthermore, there was the correlation of arterial pressure with the proinflammatory cytokines levels of blood plasma in experimental animals (21–24).

IL-10 is an important anti-inflammatory cytokine produced by Treg-cells. Treg cells from rats with mild hypertension produced more IL-10 than did those from salt-sensitive rats (25). We found an increase IL-10 in the serum of mercury-exposed residents with hypertension.

In addition to the already discussed cytokines, IL-2 has been implicated in the development of hypertension in humans and animal models. Subcutaneous injection of IL-2 to young spontaneously hypertensive rats prevented the development of hypertension and normalized blood pressure in salt-sensitive rats with stable hypertension (26). In our study, we identified an association of high mercury levels in the urine with an increased level of interleukin-2. Possibly, this detection is due to the regulatory effect of IL-2 associated with the synthesis and secretion of other cytokines (IL-4, IL-6, INF-g, CSFs, TNF-a) (26).

At present time, few studies have confirmed the effect of mercury on the content of cytokines and little has been studied of its role in the development of pathological conditions. The study of TNF-α and IL-6 levels in the blood serum of children aged 9 to 11 yr, an increase of Hg levels were associated with a shorter sleep duration and a lower TNF-α level. IL-6 was not associated with sleep or blood mercury (27). A cross-sectional study of the population in Amazonian Brazil (28) demonstrated that the levels of proinflammatory cytokines IL-1, TNF-α and IFN-γ were significantly higher in mercury-exposed subjects and anti-nucleolar autoantibodies + (AN (o) A +) as compared to participants with lower mercury exposure. The anti-inflammatory cytokine IL-10 was not highly expressed and its levels were below the threshold for most participants regardless of exposure to mercury or AN (o) A.

## Conclusion

In the north of Central Kazakhstan, despite the purification works of the Nura River, the toxicological effect of mercury persisted among the exposed participants. The toxic effects may be related to the duration of residence of the participants in the affected territory, as shown by the elevated levels of inorganic mercury in the urine of exposed participants.

Prolonged mercury exposure increases the risk of developing hypertension through systemic inflammation, as demonstrated by elevated levels of serum inflammatory cytokines TNF-α, IL-2, IL-6 and anti-inflammatory cytokine IL-10. The presence of mercury in the body probably affected the expression of interleukin-2, one of the main cytokines that coordinate immune response.

## Ethical considerations

Ethical issues (Including plagiarism, informed consent, misconduct, data fabrication and/or falsification, double publication and/or submission, redundancy, etc.) have been completely observed by the authors.

## References

[1] Tchounwou PB Yedjou CG Patlolla AK Sutton DJ (2012). Heavy metal toxicity and the environment. In Luch. A, ed. Molecular, clinical and environmental toxicology. Experienta Supplementum. vol. 101, Springer, Basel, pp. 33–164. 10.1007/978-3-7643-8340-4_6 PMC4144270 22945569

[2] Syversen T Kaur P (2012). The toxicology of mercury and its compounds. J Trace Elem Med Biol, 26(4): 215–26. 22658719 10.1016/j.jtemb.2012.02.004

[3] Clarkson TW Magos L Myers GJ (2003). The toxicology of mercury—current exposures and clinical manifestations. N Engl J Med, 349(18): 1731–7. 14585942 10.1056/NEJMra022471

[4] Houston MC (2011). Role of mercury toxicity in hypertension, cardiovascular disease, and stroke. J Clin Hypertens (Greenwich), 13(8): 621–7. 21806773 10.1111/j.1751-7176.2011.00489.x PMC8108748

[5] Fernandes Azevedo B Barros Furieri L Peçanha FM (2012). Toxic effects of mercury on the cardiovascular and central nervous systems. J Biomed Biotechnol, 2012: 949048. 22811600 10.1155/2012/949048 PMC3395437

[6] Andreoli V Sprovieri F (2017). Genetic aspects of susceptibility to mercury toxicity: an overview. Int J Environ Res Public Health, 14(1): 93. 10.3390/ijerph14010093 PMC5295343 28106810

[7] Heaven S Ilyushchenko MA Kamberov IM (2000). Mercury in the River Nura and its floodplain, Central Kazakhstan: II. Floodplain soils and riverbank silt deposits. Sci Total Environ, 260(1–3): 45–55. 11032115 10.1016/s0048-9697(00)00566-0

[8] Heaven S Ilyushchenko MA Tanton TW (2000). Mercury in the River Nura and its floodplain, Central Kazakhstan: I. River sediments and water. Sci Total Environ, 260(1–3): 35–44. 11032114 10.1016/s0048-9697(00)00540-4

[9] Hsiao HW Ullrich SM Tanton TW (2011). Burdens of mercury in residents of Temirtau, Kazakhstan: I: Hair mercury concentrations and factors of elevated hair mercury levels. Sci Total Environ, 409(11): 2272–80. 20092877 10.1016/j.scitotenv.2009.12.040

[10] Abdullah R (2013). Implementation completion and results report (IBRD-46930) on a loan in the amount of US$ 40.39 million to the government of Kazakhstan for the Nura River Clean-up Project. Sustainable Development Department, Kazakhstan, Country Unit, Europe and Central Asia Region. Document of The World Bank. Available from: https://goo.gl/5BYn84

[11] Ing. Marek Šír (2015). Contaminated sites and their management. Case studies: Kazakhstan and Armenia. Available from: https://goo.gl/afqaz7

[12] Fillion M Mergler D Passos CJ (2006). A preliminary study of mercury exposure and blood pressure in the Brazilian Amazon. Environ Health, 5(1): 29 17032453 10.1186/1476-069X-5-29 PMC1617089

[13] Yorifuji T Tsuda T Kashima S (2010). Long-term exposure to methylmercury and its effects on hypertension in Minamata. Environ Res, 110(1): 40–6. 19922910 10.1016/j.envres.2009.10.011

[14] Salonen JT Seppänen K Lakka TA (2000). Mercury accumulation and accelerated progression of carotid atherosclerosis: a population-based prospective 4-year follow-up study in men in eastern Finland. Atherosclerosis, 148(2): 265–73. 10657561 10.1016/s0021-9150(99)00272-5

[15] Valera B Dewailly É Poirier P (2009). Environmental mercury exposure and blood pressure among Nunavik Inuit adults. Hypertension, 54(5): 981–6. 19805642 10.1161/HYPERTENSIONAHA.109.135046

[16] Bautista LE Stein JH Morgan BJ (2009). Association of blood and hair mercury with blood pressure and vascular reactivity. WMJ, 108(5): 250–2. 19743756 PMC4593490

[17] Granger JP (2006). An emerging role for inflammatory cytokines in hypertension. Am J Physiol Heart Circ Physiol, 290(3): H923–4. 16467462 10.1152/ajpheart.01278.2005

[18] Chae CU Lee RT Rifai N Ridker PM (2001). Blood pressure and inflammation in apparently healthy men. Hypertension, 38(3): 399–403. 11566912 10.1161/01.hyp.38.3.399

[19] Bautista LE Vera LM Arenas IA Gamarra G (2005). Independent association between inflammatory markers (C-reactive protein, interleukin-6, and TNF-α) and essential hypertension. J Hum Hypertens, 19(2): 149–54. 15361891 10.1038/sj.jhh.1001785

[20] Yu X Yang Z Yu M (2010). Correlation of tumor necrosis factor alpha and interleukin 6 with hypertensive renal damage. Ren Fail, 32(4): 475–9. 20446787 10.3109/08860221003664280

[21] Alexander BT Cockrell KL Massey MB (2002). Tumor necrosis factor–α–induced hypertension in pregnant rats results in decreased renal neuronal nitric oxide synthase expression. Am J Hypertens, 15(2): 170–5. 11863253 10.1016/s0895-7061(01)02255-5

[22] Orshal JM Khalil RA (2004). Reduced endothelial NO-cGMP–mediated vascular relaxation and hypertension in IL-6–infused pregnant rats. Hypertension, 43(2): 434–44. 14707155 10.1161/01.HYP.0000113044.46326.98

[23] LaMarca BB Bennett WA Alexander BT (2005). Hypertension Produced by Reductions in Uterine Perfusion in the Pregnant Rat: Role of Tumor Necrosis Factor-Alpha. Hypertension, 46(4): 1022–5. 16144982 10.1161/01.HYP.0000175476.26719.36

[24] Lee DL Sturgis LC Labazi H (2006). Angiotensin II hypertension is attenuated in interleukin-6 knockout mice. Am J Physiol Heart Circ Physiol, 290(3): H935–40. 16284237 10.1152/ajpheart.00708.2005

[25] Viel EC Lemarié CA Benkirane K (2009). Immune regulation and vascular inflammation in genetic hypertension. Am J Physiol Heart Circ Physiol, 298(3): H938–44. 20044442 10.1152/ajpheart.00707.2009

[26] Lip GY Hall JE (2007). Comprehensive Hypertension. 1st ed Mosby/Elsevier 1228 p.

[27] Gump BB Gabrikova E Bendinskas K (2014). Low-level mercury in children: Associations with sleep duration and cytokines TNF-α and IL-6. Environ Res, 134: 228–32. 25173056 10.1016/j.envres.2014.07.026 PMC4262607

[28] Gardner RM Nyland JF Silva IA (2010). Mercury exposure, serum antinuclear/antinucleolar antibodies, and serum cytokine levels in mining populations in Amazonian Brazil: a cross-sectional study. Environ Res, 110(4): 345–54. 20176347 10.1016/j.envres.2010.02.001 PMC2873228

